# Impact of ^18^F-FDG-PET/CT on the identification of regional lymph node metastases and delineation of the primary tumor in esophageal squamous cell carcinoma patients

**DOI:** 10.1007/s00066-020-01630-y

**Published:** 2020-05-19

**Authors:** Stefan Münch, Lisa Marr, Benedikt Feuerecker, Hendrik Dapper, Rickmer Braren, Stephanie E. Combs, Marciana-Nona Duma

**Affiliations:** 1 Department of Radiation Oncology, Klinikum rechts der Isar, Technical University Munich, Ismaninger Str. 22, 81675 Munich, Germany; 2 Partner Site Munich, German Cancer Consortium (DKTK), Munich, Germany; 3 Department of Nuclear Medicine, Klinikum rechts der Isar, Technical University Munich, Ismaninger Str. 22, 81675 Munich, Germany; 4 Institute of Radiology, Klinikum rechts der Isar, Technical University Munich, Ismaninger Str. 22, 81675 Munich, Germany; 5 Institute of Radiation Medicine (IRM), Helmholtz Zentrum München, Ingolstädter Landstraße 1, 85764 Oberschleißheim, Germany; 6 Department of Radiation Oncology, Universitätsklinikum Jena, Friedrich-Schiller-Universität Jena, Bachstraße 18, 07743 Jena, Germany

**Keywords:** Chemoradiation, Computed tomography, Involved-field, PET-based, Pattern of lymph node metastases

## Abstract

**Purpose:**

In patients undergoing chemoradiation for esophageal squamous cell carcinoma (ESCC), the extent of elective nodal irradiation (ENI) is still discussed controversially. This study aimed to analyze patterns of lymph node metastases and their correlation with the primary tumor using ^18^F‑fludeoxyglucose positron emission tomography/computed tomography (FDG-PET/CT) scans.

**Methods:**

102 ESCC patients with pre-treatment FDG-PET/CT scans were evaluated retrospectively. After exclusion of patients with low FDG uptake and patients without FDG-PET-positive lymph node metastases (LNM), 76 patients were included in the final analysis. All LNM were assigned to 16 pre-defined anatomical regions and classified according to their position relative to the primary tumor (above, at the same height, or below the primary tumor). In addition, the longitudinal distance to the primary tumor was measured for all LNM above or below the primary tumor. The craniocaudal extent (i.e., length) of the primary tumor was measured using FDG-PET imaging (L_PET_) and also based on all other available clinical and imaging data (endoscopy, computed tomography, biopsy results) except FDG-PET (L_CT/EUS_).

**Results:**

Significantly more LNM were identified with ^18^F‑FDG-PET/CT (177 LNM) compared to CT alone (131 LNM, *p* < 0.001). The most common sites of LNM were paraesophageal (63% of patients, 37% of LNM) and paratracheal (33% of patients, 20% of LNM), while less than 5% of patients had supraclavicular, subaortic, diaphragmatic, or hilar LNM. With regard to the primary tumor, 51% of LNM were at the same height, while 25% and 24% of lymph node metastases were above and below the primary tumor, respectively. For thirty-three LNM (19%), the distance to the primary tumor was larger than 4 cm. No significant difference was seen between L_CT/EUS_ (median 6 cm) and L_PET_ (median 6 cm, *p* = 0.846)

**Conclusion:**

^18^F‑FDG-PET can help to identify subclinical lymph node metastases which are located outside of recommended radiation fields. PET-based involved-field irradiation might be the ideal compromise between small treatment volumes and decreasing the risk of undertreatment of subclinical metastatic lymph nodes and should be further evaluated.

## Introduction

Patients with locally advanced esophageal squamous cell carcinoma (ESCC) are usually treated with neoadjuvant chemoradiation and surgery (nCRT + S). This multidisciplinary approach increases the rate of complete tumor resection, overall survival (OS), and progression-free survival (PFS) compared to surgery alone [1–3]. For patients with irresectable tumors or those unfit for or declining surgery, definitive chemoradiation (dCRT) is the recommended treatment of choice [4, 5].

Over the past decades, the longitudinal safety margins in neoadjuvant or definitive chemoradiation for ESCC have been continuously reduced [1, 3, 6–8]. While the whole esophagus was irradiated in early studies [6], recent trials used longitudinal safety margins of 3–4 cm [1, 8]. In addition, recommendations regarding the coverage of regional lymphatic pathways have also changed over time. Today, elective nodal irradiation (ENI) is not recommended in case of neoadjuvant chemoradiation [1, 8], while it is still a matter of debate in case of dCRT [9]. It is obvious that the reduction of longitudinal safety margins and consideration of involved-field irradiation (IFI) requires reliable diagnostic and imaging techniques to identify the primary tumor and metastatic lymph nodes. Therefore, international guidelines recommend clinical tumor staging with endoscopy, endoscopic ultrasound (EUS), and computed tomography (CT) [4, 5]. Furthermore, ^18^F-fludeoxyglucose positron emission tomography/computed tomography (FDG-PET/CT) is often performed in order to rule out distant metastases before starting curative treatment. Because FDG-PET/CT has also demonstrated promising sensitivity, specificity, and accuracy regarding the detection of lymph node metastases as well as the detection of the primary tumor [10, 11], implementation of PET into the radiation planning process might change the resulting target volumes, as it has already been demonstrated for other tumor entities like prostate cancer or squamous cell cancer of the tongue [12, 13].

The purpose of this study was to analyze the FDG-PET/CT-based pattern of lymph node metastases, their distance from the primary tumor, and correlations with the primary tumor extension in patients with ESCC.

## Patients and methods

### Patients

Medical records and imaging information of 102 ESCC patients who underwent PET/CT for staging purposes between 2011 and 2016 were retrospectively reviewed. 5 patients (5%) without sufficient FDG uptake, of whom 4 patients had early tumor stages (Tis or T1), and 21 patients without LNM (*n* = 6) or LNM which were only seen by endoscopic ultrasound (*n* = 15) were excluded from the analysis. In the end, a total of 76 patients were included in the analysis.

Tumor location was classified according to the upper end of the primary tumor within the esophagus. Table 1 presents clinical characteristics of patients included in this analysis. In summary, the median age of patients was 67 years and 71% of patients were male. 91% of patients had locally advanced tumors (T3/4). In 9 patients (12%) the primary tumor was located in the lower third of the esophagus, while the tumor was located within the upper or middle third of the esophagus in 41 and 47% of patients, respectively.

**Table 1:** Clinical characteristics of patients included in this analysis (*n* = 76)

Age, years; median (range)	67 (41–80)
Male sex, *n* (%)	54 (71)
Tumor location	
Upper, *n* (%)	31 (41)
Middle, *n* (%)	36 (47)
Lower, *n* (%)	9 (12)
Tumor grade	
G1, *n* (%)	1 (1)
G2, *n* (%)	34 (49)
G3, *n* (%)	35 (50)
Clinical T stage	
T2, *n* (%)	7 (9)
T3, *n* (%)	65 (86)
T4, *n* (%)	4 (5)

### Lymph node metastases

Assessment of lymph nodes was based on morphology and/or FDG uptake. Thereby, reading and interpretation of PET/CT was done by at least two experienced nuclear medicine physicians and radiologists, also taking other available information such as clinical signs and patients’ symptoms, endo- and gastroscopic images and reports, and, if applicable, diagnostic CT images into account. Regions of interest (ROI) in the esophageal lesions and in suspicious lymph nodes were defined based on areas of high regional FDG uptake. ROIs were manually adjusted if areas with high physiological uptake of surrounding areas were present (e.g., the myocardium). Images were interpreted using the Syngo Workstation (Siemens, Syngo MMVVP version VE36A; Siemens Healthineers, Erlangen, Germany) and Sectra PACS (Sectra ids7, Linköping, Sweden).

In the following, all LNM were assigned to one of 16 predefined lymph node regions (right cervical, left cervical, right supraclavicular, left supraclavicular, retrosternal, right paratracheal, left paratracheal, subaortic, subcarinal, paraesophageal above the carina, paraesophageal below the carina, right hilar, left hilar, diaphragmatic, gastric, and celiac). Furthermore, all LNM were classified according to their location relative to the primary tumor (above, same height, or below). All available information (PET/CT, endoscopy, and endoscopic ultrasound [EUS]) were used to identify the primary tumor location. In case of skip metastases or more than one tumor foci, primary tumor extension was defined from the most cranial lesion to the most caudal lesion. Thereafter, the longitudinal distance between LNM and the upper primary tumor margin (for lymph nodes above the primary tumor) or the lower primary tumor margin (for lymph nodes below the primary tumor) was measured within the CT dataset.

### Primary tumor length

To analyze the impact of PET imaging on visualization of the primary tumor, two different approaches were independently pursued. At first, the length of the primary tumor was assessed using all available diagnostic information (endoscopy, computed tomography, biopsy results) except PET imaging (L_CT/EUS_). In a second approach, the length of the primary tumor was measured after rigid fusion of CT and attenuation-corrected PET scans (L_PET_).

In case of skip metastases or multiple tumor lesions, tumor length was defined as the distance from the upper margin of the most cranial lesion to the lower margin of the most caudal lesion.

### Statistics

Statistical calculations were performed using SPSS 18.0 software (SPSS Inc, Chicago, IL, USA). The distribution of quantitative data is described by mean/median and standard deviation/interquartile range (IQR). Likewise, qualitative data is presented by absolute and relative frequencies. Statistical testing was performed using the Wilcoxon signed-rank test for paired samples. The Pearson correlation coefficient was used to describe the correlation between paired samples. Statistical significance was considered at a *p*-value < 0.05.

## Results

### Lymph node metastases

In summary, 177 LNM were identified in 76 ESCC patients. Thereby, PET/CT imaging identified significantly more lymph node metastases compared with computed tomography alone (177 vs. 131 lymph node metastases, *p* < 0.001). While 122/168 (73%) LNM with enhanced FDG uptake were also defined as metastatic due to their morphology, 9/131 (7%) of morphologically suspected LNM had no increased FDG uptake.

**Fig. 1:**
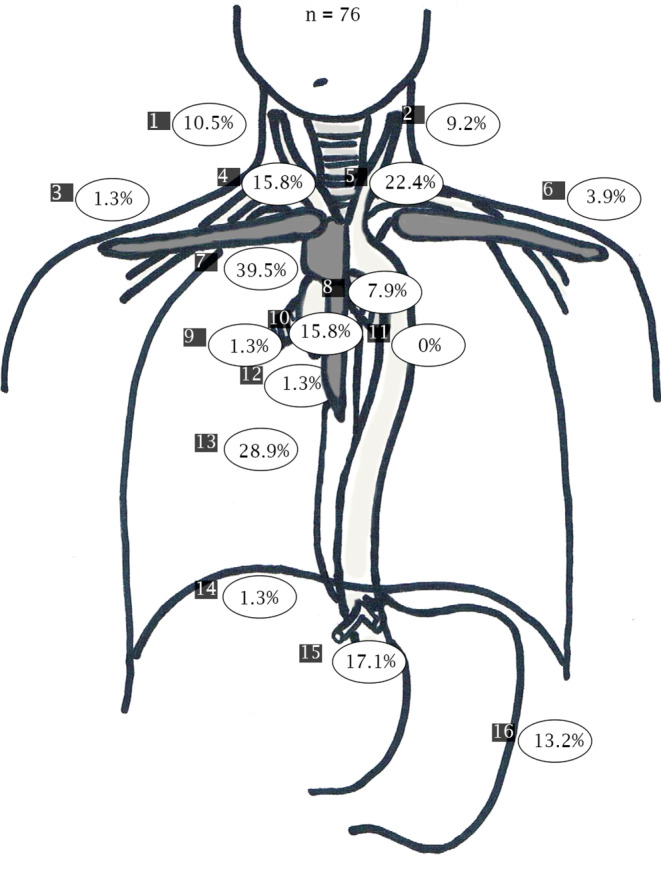
Pattern of lymph node metastases. 1. Right cervical, 2. left cervical, 3. right supraclavicular, 4. right paratracheal, 5. left paratracheal, 6. left supraclavicular, 7. paraesophageal above the carina, 8. subaortic, 9. right hilar, 10. subcarinal, 11. left hilar, 12. retrosternal, 13. paraesophageal below the carina, 14. diaphragmatic, 15. celiacal, 16. gastric

The most common sites of LNM were paraesophageal above the carina (40% of patients), paraesophageal below the carina (29% of patients), and left paratracheal (22% of patients), while the least common sites of LNM in the whole patient cohort were supraclavicular, hilar, diaphragmatic, and retrosternal (<5% of patients; Fig. 1).

**Fig. 2:**
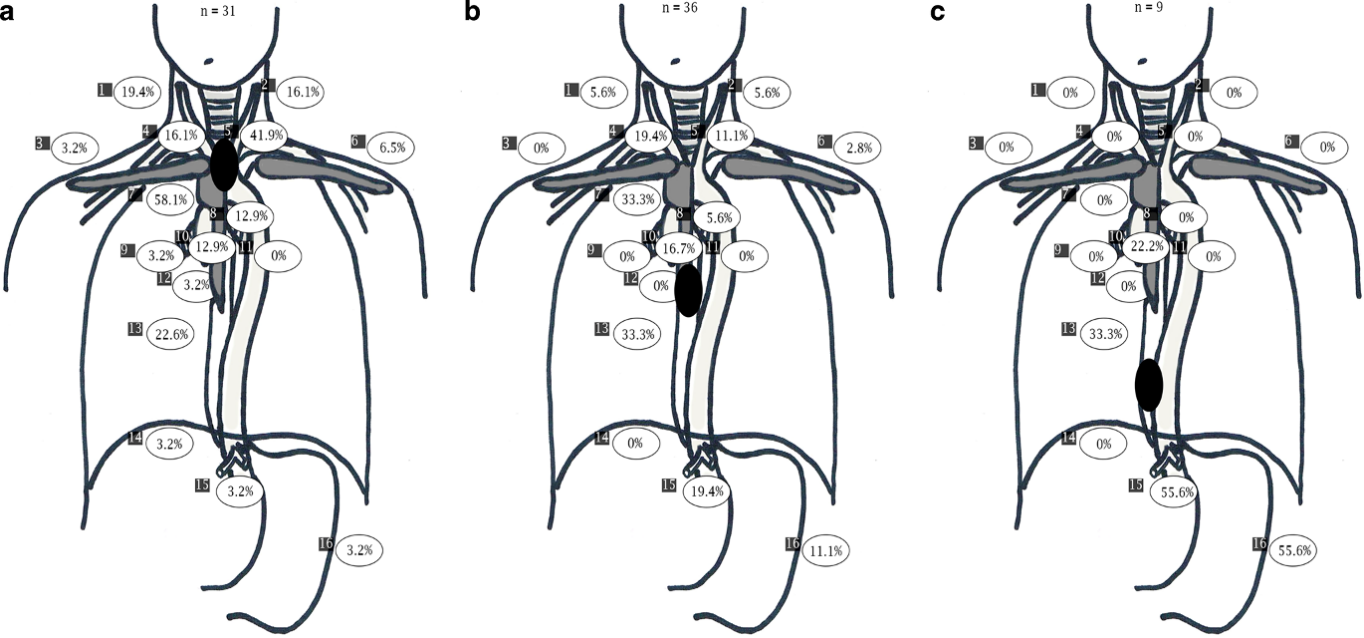
Pattern of lymph node metastases depending on the location of the primary tumor. **a** Patients with a primary tumor in the upper third of the esophagus. **b** Patients with a primary tumor in the middle third of the esophagus. **c** Patients with a primary tumor in the lower third of the esophagus. 1. Right cervical, 2. left cervical, 3. right supraclavicular, 4. right paratracheal, 5. left paratracheal, 6. left supraclavicular, 7. paraesophageal above the carina, 8. subaortic, 9. right hilar, l0. subcarinal, 11. left hilar, 12. retrosternal, 13. paraesophageal below the carina, 14. diaphragmatic, 15. celiac, 16. gastric

For patients with tumors in the upper third of the esophagus (*n* = 31), the most common sites of LNM were paraesophageal above the carina (58% of patients) and left paratracheal (42% of patients). Less than 5% of these patients had LNM right supraclavicular, gastric, diaphragmatic, celiac, hilar, and retrosternal. In contrast, for patients with primary tumors in the middle third of the esophagus (*n* = 36), patients most commonly presented with paraesophageal LNM above (33% of patients) and below the carina (33% of patients), but patients rarely presented with supraclavicular, retrosternal, gastric, or hilar LNM (<5% of patients). Within the subgroup of patients with primary tumors in the lower third of the esophagus (*n* = 9), LNM were seen in the gastric region (56% of patients), celiac (56% of patients), paraesophageal below the carina (33% of patients), and subcarinal (22% of patients; Fig. 2a–c).

**Fig. 3:**
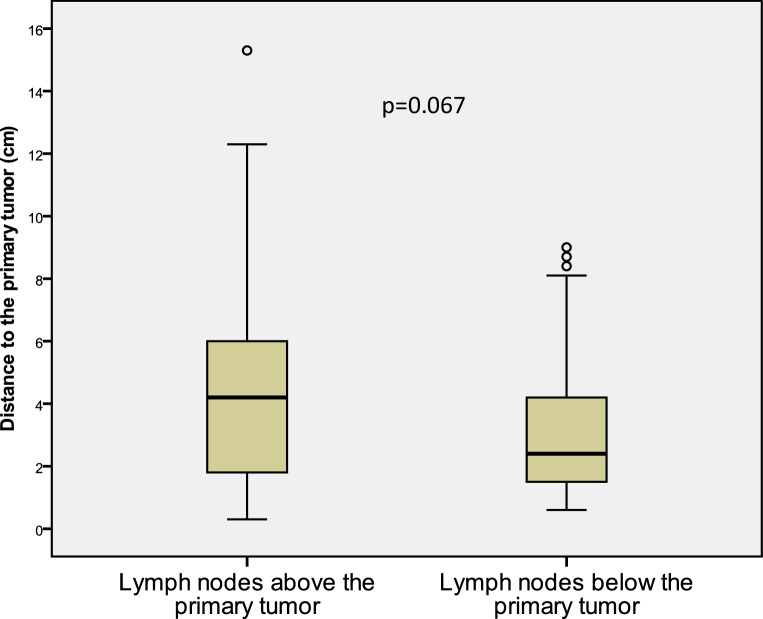
Classical boxplot demonstrating the distance of lymph node metastases to the primary tumor

With regard to the location of the primary tumor, 51% (*n* = 90) of LNM were at the height of the primary tumor, 25% (*n* = 45) of LNM were above the primary tumor, and 24% (*n* = 42) were below the primary tumor. The median distance of LNM to the primary tumor was 4.2 and 2.4 cm (*p* = 0.067) for LNM above the primary tumor and LNM below the primary tumor, respectively. For 33 LNM (19%), the distance to the primary tumor was >4 cm (Fig. 3).

### Primary tumor

**Fig. 4:**
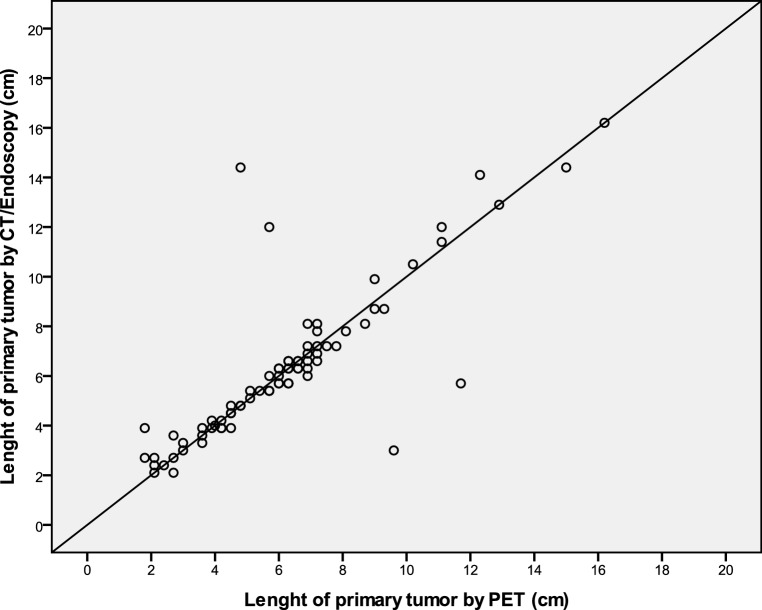
Length of the primary tumor as assessed by CT/endoscopy and PET. *CT* computed tomography, *PET* positron-emission tomography

No significant difference was seen between L_CT/EUS_ (median tumor length 6 cm, IQR 4–7.2 cm) and L_PET_ (median tumor length 6 cm, IQR 4.2–7.2 cm, *p* = 0.846). In addition, a significant and strong correlation was seen between L_CT/EUS_ and L_PET_ (r = 0.830; Fig. 4).

An absolute difference of more than or equal to 1 cm between L_PET_ and L_CT/EUS_ was seen in 7 patients (9%). Thereby, L_CT/EUS_ was longer than L_PET_ in 5 patients (71%).

## Discussion

We evaluated the FDG-PET/CT-based pattern of lymph node metastases and their distance to the primary tumor in patients with ESCC. PET/CT was able to identify significantly more lymph node metastases than CT alone, with most LNM located paraesophageally or paratracheally. Approximately half of LNM are located above or below the primary tumor, with a median distance to the primary tumor of 4.2 and 2.4 cm, respectively. No difference in terms of visualization of the primary tumor length was seen in this study.

The sensitivity of PET for identification of LNM varies in the literature, but it is higher than that of CT [10]. Furthermore, the specificity and positive predictive value of PET are approximately 90% [10, 14, 15]. Thus, there is only a small risk of false-positive lymph nodes. In addition, when using PET, most (57%) false-positive lymph nodes are seen in the hilar region due to granulomatous diseases [16]. In our study, only two LNM (1.3%) were located in the hilar region. This low rate of hilar LNM within this region might be explained by the assessment of two experienced nuclear physicians and radiologists for our study already taking this information into account for image interpretation.

Leong et al. [17] also compared delineation of primary tumor and lymph node metastases based on PET/CT or CT alone in 21 esophageal cancer patients. When considering PET/CT imaging, unsuspected lymph node metastases were detected in 4 additional patients compared to CT alone.

In our study, most LNM were located paratracheally and paraesophageally, and the pattern of LNM depended on primary tumor location, which is in line with the results of other trials [18–20]. Garcia and colleagues [18] analyzed patterns of FDG-avid lymph nodes in 473 esophageal cancer patients. In contrast to our study, these authors included patients with adenocarcinoma (71%) and squamous cell carcinoma (29%). They found that patients with upper thoracic tumors, which were defined as tumors above the carina, most often had lymph nodes in the supraclavicular (27%), upper paratracheal (11%), lower paratracheal (13%), and retrotracheal (17%) regions. In addition, cervical LNM were seen in 5% of patients. The rate of paratracheal LNM is difficult to compare between the studies because the authors did not differ between LNM located paratracheally and paraesophageally above the carina. However, in patients with upper thoracic tumors, only a small number of LNM were located below the carina (18%), which seems to be comparable to our results. In patients with lower thoracic tumors, in the study of Garcia et al., the most commonly involved lymph nodes were located paraesophageally below the carina (19%) and abdominally (54%). Although we have to keep in mind that most of these tumors (80%) were adenocarcinoma, this is also in line with our results and the knowledge of the lymphatic spread of the lower esophagus.

Garcia et al. [18] further analyzed the distance of LNM to the primary tumor: 11% (for upper thoracic tumors) and 58% (for lower thoracic tumors) of the paraesophageal lymph nodes were non-adjacent to the primary tumor. In our study, 89% of patients had tumors in the upper or middle third of the esophagus and 49% of LNM were non-adjacent. This lower rate of non-adjacent LNM described by Garcia et al. is probably explained by the fact that distance to the primary tumor was only assessed for paraesophageal lymph nodes. In addition, the median distance between LNM and the primary tumor was 0 cm for upper thoracic tumors and 1.5 cm for lower thoracic tumors, which is smaller than in our study. However, this difference is explained by the fact that the distance to the primary tumor was calculated by including all lymph nodes in the study by Garcia et al., while in our study the median distance was calculated for LNM above and below the primary tumor only. This approach is necessary in order to determine whether the craniocaudal safety margins are adequate to cover occult paraesophageal nodal spread. In our opinion, the fact that the median distance to the primary tumor was higher for LMN above the primary tumor than for LNM below the primary tumor in our study (4.2 vs. 2.4 cm) might be explained by the lymphatic drainage along the thoracic duct toward the venous angle.

While many studies report a significant decrease in primary tumor length when using PET/CT compared to CT alone [21–23], the consideration of PET imaging in our study did not lead to significant changes in primary tumor length. However, in contrast to the studies mentioned above, we compared tumor length assessed by PET with tumor length assessed by the combined information of CT and EUS. Because PET strongly correlates with EUS and histopathologic results [24, 25], this probably also explains the strong correlation between tumor length as assessed by PET and CT/endoscopy in our study.

It has already been demonstrated that pretherapeutic staging using PET/CT imaging is associated with prolonged recurrence-free survival (RFS) in ESCC patients undergoing neoadjuvant or definitive chemoradiation [26]. Since PET imaging increases the detection rate of distant metastases compared to CT [27, 28], some patients who are only staged with CT might have undetected distant metastases which affect RFS. However, based on our results, one could speculate that consideration of PET imaging for radiation planning also improves RFS by identifying metastatic lymph nodes which are located outside of standard PTVs. In the already mentioned study by Leong and colleagues [17], morphologically unsuspected lymph nodes with pathologic FDG uptake which were also located outside the CT-based treatment volume were identified in 3 of 21 patients (14%).

The omission of ENI not only revealed promising results in case of neoadjuvant chemoradiation [1], but there was also no difference regarding OS or local control between ENI and IFI in case of definite chemoradiation [9, 29]. In addition, IFI can reduce the dose distribution to the lungs and the rate of pulmonary toxicities [29, 30], while a concurrent reduction of the doses to the heart can decrease the rate of symptomatic or asymptomatic pericardial effusion [31]. This should be kept in mind before considering ENI, even in patients who did not undergo PET staging. Nonetheless, in 20% of our patients, some LNM were more than 4 cm away from the primary tumor.

This study has some limitations. By focusing on PET/CT imaging to identify LNM, small peritumoral lymph nodes might be missed in some patients. This assumption is confirmed by the fact that PET/CT only identified LNM in 76 of 91 patients (84%) who were staged as N+ by EUS. This is probably caused by the limited spatial resolution of PET. Therefore, the rate of peritumoral paraesophageal LNM might be underestimated in this study. Since this is only a problem for LNM adjacent to the primary tumor, the rate of LNM located at the same height as the primary tumor might also be underestimated. Nonetheless, these lymph nodes do not represent a problem in the planning process due to the circular safety margin. Another limitation is that LNM were identified by PET/CT imaging only. As discussed earlier, this bears the risk of including false-positive LNM. However, given the high positive predictive value of up to 93% for regional LNM [14, 15], this effect should be limited. In this context, we should also state that due to the retrospective nature of the study, no standardized SUVmax value was used to identify LNM, which impacts the comparability and also affects sensitivity and specificity. Nonetheless, all imaging data were assessed by experienced nuclear physicians and/or radiologists.

In conclusion, ^18^F‑FDG-PET can help to identify subclinical lymph node metastases which are located outside of recommended radiation fields. PET-based involved-field irradiation might be the ideal compromise between small treatment volumes and decreasing the risk of undertreatment of subclinical metastatic lymph nodes and should be further evaluated.

## Abbreviations

dCRT: Definitive chemoradiation
ENI: Elective nodal irradiation
ESCC: Esophageal squamous cell carcinoma
EUS: Endoscopic ultrasound
FDG: ^18^F‑fludeoxyglucose
IFI: Involved-field irradiation
L_CT/EUS_: Tumor lengths assessed by CT and endoscopy
LNM: Lymph node metastases
L_PET_: Tumor length assessed by PET
nCRT + S: Neoadjuvant chemoradiation and surgery
OS: Overall survival
PET/CT: Positron emission tomography/computed tomography
PFS: Progression-free survival
RFS: Recurrence-free survival

## References

[1] van Hagen P (2012). Preoperative chemoradiotherapy for esophageal or junctional cancer. N Engl J Med.

[2] Shapiro J (2015). Neoadjuvant chemoradiotherapy plus surgery versus surgery alone for oesophageal or junctional cancer (CROSS): long-term results of a randomised controlled trial. Lancet Oncol.

[3] Tepper J (2008). Phase III trial of trimodality therapy with cisplatin, fluorouracil, radiotherapy, and surgery compared with surgery alone for esophageal cancer: CALGB 9781. J Clin Oncol.

[4] Porschen R (2019). S3-Leitlinie – Diagnostik und Therapie der Plattenepithelkarzinome und Adenokarzinome des Ösophagus, Langversion 2.0, 2018. AWMF Registernummer: 021/023OL. Z Gastroenterol.

[5] National Comprehensive Cancer Network (2019) Clinical practise guidelines in oncology—esophageal and esophagogastric junction cancers. https://www.nccn.org/professionals/physician_gls/pdf/esophageal.pdf. Accessed 5 Jan 2020

[6] Herskovic A (1992). Combined chemotherapy and radiotherapy compared with radiotherapy alone in patients with cancer of the esophagus. N Engl J Med.

[7] Minsky BD (2002). INT 0123 (Radiation Therapy Oncology Group 94-05) phase III trial of combined-modality therapy for esophageal cancer: high-dose versus standard-dose radiation therapy. J Clin Oncol.

[8] Yang H (2018). Neoadjuvant chemoradiotherapy followed by surgery versus surgery alone for locally advanced squamous cell carcinoma of the esophagus (NEOCRTEC5010): a phase III multicenter, randomized, open-label clinical trial. J Clin Oncol.

[9] Wang X (2017). Can involved-field irradiation replace elective nodal irradiation in chemoradiotherapy for esophageal cancer? A systematic review and meta-analysis. Onco Targets Ther.

[10] Muijs CT (2010). A systematic review on the role of FDG-PET/CT in tumour delineation and radiotherapy planning in patients with esophageal cancer. Radiother Oncol.

[11] Choi JY (2000). Improved detection of individual nodal involvement in squamous cell carcinoma of the esophagus by FDG PET. J Nucl Med.

[12] Walacides D (2019). Comparison of (68)Ga-PSMA ligand PET/CT versus conventional cross-sectional imaging for target volume delineation for metastasis-directed radiotherapy for metachronous lymph node metastases from prostate cancer. Strahlenther Onkol.

[13] Samolyk-Kogaczewska N (2019). PET/MRI-guided GTV delineation during radiotherapy planning in patients with squamous cell carcinoma of the tongue. Strahlenther Onkol.

[14] Okada M (2009). Integrated FDG-PET/CT compared with intravenous contrast-enhanced CT for evaluation of metastatic regional lymph nodes in patients with resectable early stage esophageal cancer. Ann Nucl Med.

[15] Shen H (2012). Confirmation of histology of PET positive lymph nodes recovered by hand-video-assisted thoracoscopy surgery. Gene.

[16] Yoon YC (2003). Metastasis to regional lymph nodes in patients with esophageal squamous cell carcinoma: CT versus FDG PET for presurgical detection prospective study. Radiology.

[17] Leong T (2006). A prospective study to evaluate the impact of FDG-PET on CT-based radiotherapy treatment planning for oesophageal cancer. Radiother Oncol.

[18] Garcia B (2016). Distribution of FDG-avid nodes in esophageal cancer: implications for radiotherapy target delineation. Radiat Oncol.

[19] Huang W (2010). Pattern of lymph node metastases and its implication in radiotherapeutic clinical target volume in patients with thoracic esophageal squamous cell carcinoma: a report of 1077 cases. Radiother Oncol.

[20] Zhang J (2018). Pattern of lymph node metastasis in thoracic esophageal squamous cell carcinoma with poor differentiation. Mol Clin Oncol.

[21] Muijs CT (2009). Consequences of additional use of PET information for target volume delineation and radiotherapy dose distribution for esophageal cancer. Radiother Oncol.

[22] Jimenez-Jimenez E (2018). Radiotherapy volume delineation using 18F-FDG-PET/CT modifies gross node volume in patients with oesophageal cancer. Clin Transl Oncol.

[23] Konski A (2005). The integration of 18-fluoro-deoxy-glucose positron emission tomography and endoscopic ultrasound in the treatment-planning process for esophageal carcinoma. Int J Radiat Oncol Biol Phys.

[24] Mamede M (2007). Pre-operative estimation of esophageal tumor metabolic length in FDG-PET images with surgical pathology confirmation. Ann Nucl Med.

[25] Jeganathan R (2011). Does pre-operative estimation of oesophageal tumour metabolic length using 18F-fluorodeoxyglucose PET/CT images compare with surgical pathology length?. Eur J Nucl Med Mol Imaging.

[26] Metzger JC (2017). Inclusion of PET-CT into planning of primary or neoadjuvant chemoradiotherapy of esophageal cancer improves prognosis. Strahlenther Onkol.

[27] Heeren PA (2004). Detection of distant metastases in esophageal cancer with (18)F-FDG PET. J Nucl Med.

[28] Flamen P (2000). Utility of positron emission tomography for the staging of patients with potentially operable esophageal carcinoma. J Clin Oncol.

[29] Cheng YJ (2018). Comparison of elective nodal irradiation and involved-field irradiation in esophageal squamous cell carcinoma: a meta-analysis. J Radiat Res.

[30] Li DJ (2016). Patterns of failure after involved field radiotherapy for locally advanced esophageal squamous cell carcinoma. J BUON.

[31] Ogino I (2017). Dosimetric predictors of radiation-induced pericardial effusion in esophageal cancer. Strahlenther Onkol.

